# Exploring the Experiences and Perspectives of Insulin Therapy in Type 2 Diabetes via Web-Based UK Diabetes Health Forums: Qualitative Thematic Analysis of Threads

**DOI:** 10.2196/34650

**Published:** 2022-10-05

**Authors:** Maya Allen-Taylor, Laura Ryan, Kirsty Winkley, Rebecca Upsher

**Affiliations:** 1 Florence Nightingale Faculty of Nursing Midwifery and Palliative Care King's College London London United Kingdom; 2 Institute of Psychiatry, Psychology & Neuroscience King's College London London United Kingdom

**Keywords:** type 2 diabetes, adults, qualitative, insulin, internet, web-based

## Abstract

**Background:**

Despite the advent of type 2 diabetes (T2D) remission strategies and novel therapeutic agents, many individuals with T2D will require insulin treatment to achieve target glycemia, with the aim of preventing or delaying diabetes complications. However, insulin refusal and cessation of treatment in this group are common, and their needs are underreported and relatively unexplored.

**Objective:**

This study aimed to explore the experiences and perspectives of individuals with T2D for whom insulin therapy is indicated as expressed on web-based health forums, in order to inform the development of evidence-based structured educational and support strategies and improve health care provider awareness.

**Methods:**

Retrospective archived forum threads from the 2 largest, freely and publicly accessible diabetes health forums in the United Kingdom were screened over a 12-month period (August 2019-2020). The Diabetes UK and Diabetes.co.uk forums were searched for relevant threads. A total of 3 independent researchers analyzed the forum threads and posts via thematic analysis. Pertinent themes were identified and illustrated by paraphrasing members’ quotes to ensure anonymity. A total of 299 posts from 29 threads from Diabetes UK and 295 posts from 28 threads Diabetes.co.uk were analyzed over the study period. In all, 57 threads met the inclusion criteria and were included in the final analysis.

**Results:**

Four overarching themes were generated to illustrate the unmet needs that prompted members to seek information, advice, and support regarding insulin therapy outside of their usual care provision via the forums: empowerment through sharing self-management strategies, seeking and providing extended lifestyle advice, relationships with health care professionals, and a source of psychological peer support.

**Conclusions:**

This is the first study to collect data from web-based health forums to characterize the experiences and perspectives of people with T2D for whom insulin therapy is indicated. The observed naturalistic conversations have generated useful insights; our findings suggest that there are significant unmet self-management and psychological needs within this group that are not being met elsewhere, prompting the seeking of information and support on the web. These include practical aspects such as insulin injection technique, storage and dose titration, driving and travel considerations, the emerging use of technology, and a strong interest in the effects of extended lifestyle (diet and activity) approaches to support insulin therapy. In addition, problematic relationships with health care professionals appear to be a barrier to effective insulin therapy for some. In contrast, seeking and offering mutually beneficial, practical, and psychological support from peers was viewed as enabling. The study results will help to directly inform insulin-focused self-management and support strategies to enable individuals in this group to achieve their best outcomes.

## Introduction

### Background

Type 2 diabetes (T2D) is a significant public health challenge worldwide. In the United Kingdom, the prevalence rate of 3.9 million diagnosed individuals, plus an additional 1 million undiagnosed and unaware, is predicted to rise to 5.3 million by 2025, potentially affecting over 11% of the population [1]. The treatment of largely preventable long-term complications of T2D, including cardiovascular disease, nephropathy, retinopathy, and neuropathy, already uses 11% of the National Health Service (NHS) budget. This figure is set to rise, mirroring prevalence rates as well as the cost of increasingly expensive diabetes therapeutic agents. In addition, T2D can place a huge burden on affected individuals, their families, and society; for example, loss of income, use of social security benefits, and early retirement [2].

Effective treatment of T2D can help prevent or delay the onset of complications [3]. However, to achieve target glycemia and glycated hemoglobin (HbA1c), individuals are often required to adopt extensive self-management behaviors, such as attention to diet, activity, and adherence to multiple therapeutic agents, which may include insulin therapy. Recent developments such as diet based “remission,” and the advent of pharmacological agents such as glucagon-like peptide-1 and sodium-glucose co-transporter-2 classes have highlighted the potential to prevent or delay the requirement for insulin for some individuals [4,5]. However, for many, T2D will remain a lifelong and progressive condition, advancing from the insulin-resistant state to pancreatic beta-cell exhaustion and insulin insufficiency [6]. Mirroring these prevalence rates, the estimated number of people with T2D who were insulin-treated increased from 136,800 (95% CI 120,700-155,200) in 1991 to 421,300 (95% CI 399,800-444,100) in 2010 in the United Kingdom [7].

### Lifestyle and Therapy Challenges With Insulin in T2D

The multifaceted challenges of insulin therapy for people with T2D are complex. Current worldwide literature suggests that 25% to 47% of individuals with T2D refuse or are unwilling to start insulin [8-11]. Negative health beliefs (cognitions) are common, including fear of injections, uncertainty around efficacy, fears around hypoglycemia, potential weight gain and misconceptions that starting insulin represents a poorer prognosis, or “the end of the road” in their treatment and condition [12,13]. In total, 20% of individuals who start insulin disrupt their treatment (omitting doses) [13], and 20% to 40% cease treatment altogether [14-17]. One in 5 who do persist are affected by “diabetes distress,” an emotional state characterized by feelings of frustration, defeat or being overwhelmed [18]. In addition, health care professionals (HCPs) may perpetuate these cognitions by demonstrating “clinical inertia,” an aversion or delay in recommending insulin therapy. Collectively, these phenomena have been termed “psychological insulin resistance” [9,11].

### Diabetes Self-Management Education and Support in Insulin Therapy

Diabetes Self-Management Education and Support (DSMES) is a strategy that can be used to empower and support insulin-treated individuals with T2D. Most UK-based programs are developed and led by diabetes specialist nurses and diabetes dietitians. However, they vary in structure and curriculum, and their efficacy is limited, fractured, and rarely evidence based. A meta-analysis of insulin DSMES interventions for adults with T2D from our research group suggested a small significant reduction in HbA1c levels (N=10, standardized mean difference 0.22, 95% CI 0.34-0.10, *P*<.001). The methodological quality was moderate to poor for most studies, and the theory and evidence bases for the interventions were not well described [19]. Therefore, there is a need for effective evidence-based DSMES for insulin-treated people with T2D.

### Using the Internet for Research

A total of 92% of adults in the United Kingdom were recent internet users in 2020 [20] and 70% of people using the internet have searched for health information on the web [21]. Web-based forums are increasingly accessed by the web-based “diabetes community” to seek information, support, and discuss their concerns [22]. By joining a diabetes health forum, members seek to improve their ability to understand the condition, treatment, and improve their self-management skills, while being presented with opportunities for peer support, reassurance, and friendship [23]. Previous studies on health forums have suggested benefits from these web-based interactions, resulting in better knowledge about their condition and improved “health activation” in members [24]. To date, the web-based diabetes community in the United Kingdom has been dominated by individuals with type 1 diabetes (T1D), or parents of children with T1D. It could be argued that this group may have specific demographics that are more likely to seek health advice and support on the web, being generally younger and more activated to achieve health goals, particularly around insulin therapy. This is reflected in the number of replies from people with T1D to the posts we examined. However, the number of people with T2D who currently access the forums is significant and increasing, reflecting the growing prevalence rates, diagnosis of T2D at a younger age and increased need for insulin treatment in this group. This study is the first to explore the needs of individuals with T2D who are recommended or prescribed insulin therapy, via a thematic analysis of diabetes health forums. The threads and posts that chart personal experiences within these forums contain a wealth of information and an opportunity for researchers to examine real-world experiences [25].

## Methods

### Overview

Accessing health forums for research allows for the examination of rich data and the subjective experiences of “members” (individuals who join the forum and may post or reply on the web). Analyzing interactions between peers in this way has the potential to provide a perspective and understanding that is difficult to achieve in offline contexts and may have an advantage over other qualitative methodologies; for example, a reduction in social desirability bias [26]. This unique approach may uncover additional data that traditional qualitative methodologies may not, providing a valuable contribution to the existing body of knowledge.

### Patient and Public Involvement

A recent qualitative study published by the study team used semistructured interviews with participants who had attended a traditional, non–evidence-based insulin initiation group. Positive experiences were associated with sharing experiences with peers, reassurance, and the skill of the facilitator in addressing both practical and psychological concerns [27]. However, a subsequent patient and public involvement group with 8 insulin-treated participants with T2D suggested that there were many additional unmet needs that the study had not identified. In addition, the patient and public involvement group consensus was that they (people with T2D receiving insulin therapy) felt neglected or “the poor cousins” in terms of the current research focus on diabetes, which they felt revolved around diabetes remission or newer therapeutic agents. It became clear that an understanding of the perspectives and needs of a significant population of people with T2D who are recommended or prescribed insulin therapy or will be in the future remains significantly lacking.

### Ethics Approval

Minimal risk ethical approval for the study was granted from King’s College London University (ref: MRA-19/20-20587). Important ethical questions related to intrusiveness and perceptions of a forum as a public or private domain were considered. Data that are freely and publicly accessible, particularly if carried out “passively,” that is, the researchers do not involve themselves in the forums, have been documented as ethical. As such, no interaction with the forum was made, in keeping with observing social responsibility and the British Psychological Association code of Human Research Ethics guidelines [28]. As the data were collected from open-access websites that are in the public domain, consent was not sought. Although we have chosen to disclose the names of the forums, care has been taken to maintain the anonymity of member’s identity from the “threads” (a discussion, usually starting with a question from a member) and “posts” (questions and replies within a thread).

### Design

Two diabetes health forums were identified and selected via the 3 most popular UK internet search engines (Google, Bing, and Yahoo), accessed by 98.83% of users in 2019 to 2020 [29]. Diabetes.co.uk [30], a patient-focused, self-help website with nearly 343,000 members, is considered the most actively used social media forum for people with diabetes [31]. Diabetes UK, a charity, attracted over half a million visits to their forum in 2019 [32]. Both sites are nonsubscription, moderated (screened by the organization to ensure that only appropriate content is posted), and publicly accessible. There are other nonsubscription, web-based diabetes forums in the United Kingdom; however, these large forums were selected because of their reach, popularity, and dedicated message boards [33].

### Data Collection

**Figure 1:**
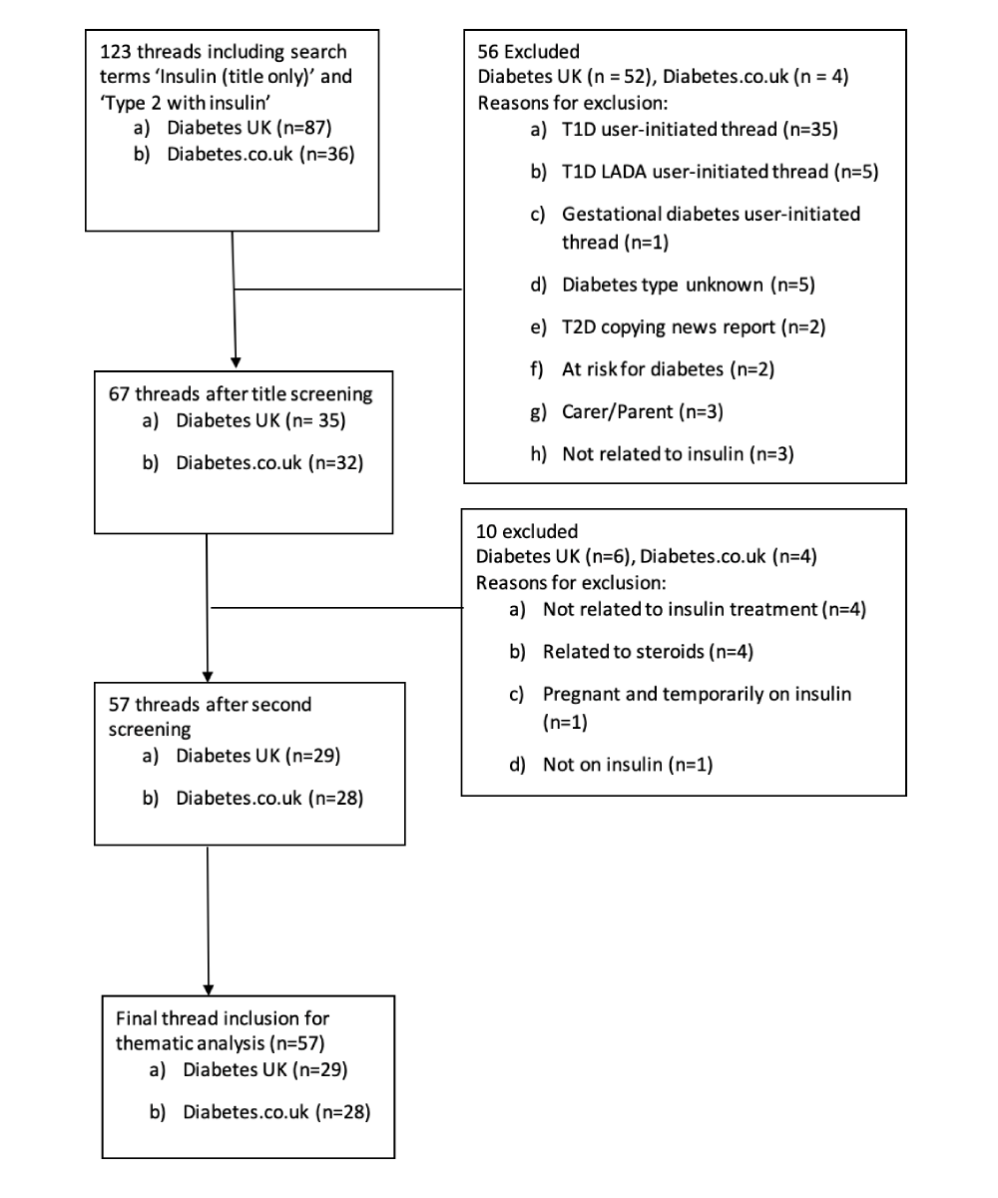
Flow of forum thread selection. T1D=Type 1 Diabetes; T2D=Type 2 Diabetes; LADA=Latent autoimmune diabetes in adults.

Retrospective archived forum threads were screened between August 1, 2019, and August 1, 2020 (ie, a 12-month period). Within the Diabetes UK forum, the search term “insulin (title only)” was used. On Diabetes.co.uk [30], threads were screened within an existing “Type 2 with insulin” message board. In the first screening stage, 2 researchers (MAT and RU) independently reviewed the titles and thread descriptions to determine eligibility. These were compared among the researchers, and any uncertainty at this stage resulted in the titles being carried forward to the next screening phase. In the second screening stage, the posts and threads were reviewed. The initial post was screened to determine relevance, that is, it was posted by a person with T2D, and was regarding insulin therapy. All eligible threads were included and unrestricted by length. For both forums, members’ self-reported information linked to each post indicated which type of diabetes the member lived with; that is, T2D, T1D, other types of diabetes, or a caregiver of someone with diabetes. Owing to a large number of replies from people with self-reported T1D, these threads were included in the final analysis. Any disagreements over final inclusion were discussed with a third researcher (KWB), presented in Figure 1. The 57 final threads were extracted and imported into individual word documents to aid analysis. Additional information was extracted for each thread: title, date posted, general thread topic, number of replies, and frequency of replies by diabetes type. Demographics, such as age and sex, were not collected, as it was not possible to corroborate this information due to the forums’ use of anonymity and self-selected member usernames. It was not possible to determine if individual members posted factual information or multiple posts under alternative usernames; however, this was recognized as a possible methodological limitation.

### Data Analysis

All posts in relevant threads were analyzed. Identifying characteristics, such as usernames, were removed and replaced with a pseudonym to protect members’ identities. Two researchers (MAT and RU) extracted the initial codes. NVivo 12 (QSR International) software was subsequently used to organize the data [34]. The data was analyzed using thematic analysis as described by Clarke and Braun [35]. Three researchers (MAT, RU, and LR) read and reread the threads to ensure thorough comprehension. The initial codes were discussed and agreed upon. The codes were subsequently compared and grouped into 4 broad themes reflecting content and intent. To illustrate the themes, extracts from across the data sources with similar content were identified. Paraphrasing members’ posts was used to ensure anonymity, which could have been compromised through the use of direct quotes. We considered the data extracts as observations of naturalistic conversations and interpreted them accordingly. The quotes were presented as a representation of the initial posts and illustrative replies.

## Results

### Overview

We identified 299 posts from 29 threads from Diabetes UK and 295 posts from 28 threads from Diabetes.co.uk [30] during the study period. A total of 57 threads met the inclusion criteria and were included in the thematic analysis after the second screening. More threads were identified via the Diabetes UK forum (n=87) than Diabetes.co.uk [30] (n=36).

In response to an initial post from a person with T2D, most replies in Diabetes UK threads were from people with T1D (155/299, 51.8%), and most replies from Diabetes.co.uk [30] threads were from people with T2D (189/295, 64.1%; Table 1).

**Table 1:** Number of replies and diabetes type of replies.

Forum member diabetes type	Diabetes UK replies (n=299), n (%)	Diabetes.co.uk [30] replies (n=295), n (%)
T2D^a^	111 (37.1)	189 (64.1)
T1D^b^	155 (51.8)	48 (16.3)
Carer or parent	9 (3)	1 (0.3)
Unknown	0 (0)	37 (12.5)

^a^T2D: type 2 diabetes.

^b^T1D: type 1 diabetes.

Following data familiarization, 91 codes were iteratively created and discussed by MAT and RU. Using the research team’s diabetes nursing, clinical psychology, academic and qualitative research experience, the codes were collated collaboratively by MAT, RU, and LR. No significant discrepancies were established. After a final discussion with KWB, 4 main themes were agreed upon: empowerment through sharing self-management strategies, seeking and providing lifestyle advice, relationships with HCPs, and a source of emotional and peer support.

### Empowerment Through Sharing of Self-management Strategies

Several threads addressed relatively simple unmet practical needs, such as correct insulin storage and technique, indicating a lack of basic self-management skills or knowledge:

Post: So much kit, where do I store it all?

Reply: All my daily supplies are in a pencil case, everything else I keep stored away and unopened insulin stays the fridge.

DC7

Post: Is it OK to have bubbles in the pens (insulin devices)?

Reply: Tap the pen...keeping it vertical...dial up a couple of units and press the plunger, hey presto!

DUK05

While others expressed that they felt almost embarrassed at their lack of knowledge:

Post: Just realised I didn’t take my insulin—not sure what to do, take it now or wait until tomorrow? Silly I know but still getting used to it.

DC8

In addition, members sought advanced self-management skills such as insulin self-titration; that is, the confidence to increase and decrease insulin doses in response to personal glucose targets. However, most members were unwilling to advise on dose increases, fearful that this would constitute “prescribing,” revealing a limitation of the forum:

Post: I’ve been on 10 units of insulin for a while now but my glucose levels are still high....should I increase the dose?

Reply: No one here on the forum can prescribe for you.

This prompted an emotive response:

Reply: I am obviously not asking for anyone to prescribe for me and I wouldn’t even if they did! I just really need some help and was hoping someone might share their experiences with me.

DC8

Driving when insulin-treated comes with specific requirements, such as informing the driving licensing authority, monitoring glucose levels regularly while driving and keeping hypoglycemia treatment in the vehicle. Failure to do so can not only be dangerous in the event of hypoglycemia but can also lead to a driving suspension or a fine. However, some members were clearly not offered appropriate education and guidance and were shocked when they read other’s posts:

Reply: I didn’t know I had to do all this! Neither my nurse nor my GP informed me when I switched to insulin nearly a year ago!

DUK30

Similarly, some members were unaware of key considerations when traveling, arguably vital information as insulin can be rendered unusable by becoming too hot or cold, leaving the person medically vulnerable, particularly when traveling overseas:

Post: I am going away, how on earth and I supposed to keep this insulin cool for 2 weeks...what do I do when I go through security with all these needles and things?

Reply: We use the FRÍO bag [provides website details] if we are in a hot place and carry a medical letter to show at security, so we are not worried about traveling with insulin anymore.

DUK3

Some members, particularly those with T1D, were keen to recommend using continuous glucose monitoring technology, despite it being generally unavailable to people with T2D via the NHS:

It’s always easier if you have Flash monitor [continuous glucose monitor], less finger pricking and better picture.

DC27

### Seeking and Providing Lifestyle Advice

Successful diabetes self-management often requires individuals to adapt their lifestyle behaviors (diet and activity). This was brought into sharper focus when starting insulin treatment. There was a clear desire and need for extended advice regarding the perceived benefits of specific diets. A low carbohydrate diet was most likely to be discussed and recommended by members, despite not being recommended by most HCPs:

Personally, I prefer to eat low carb...keeps the dose of insulin and risk of hypos [hypoglycemia] low, helps you lose weight and takes the pressure off your pancreas.

DUK10

In addition, carbohydrate counting was discussed and often extolled. Traditionally, a method used by people with T1D to calculate insulin doses in response to the carbohydrate content of food consumed, it is becoming more popular with people with T2D who are insulin-treated:

Count them [carbohydrates] and adjust your insulin accordingly, try cutting down on the carbs, all of them turn to glucose once ingested.

DUK35

I’m self taught...I can tell you, it made Christmas day far easier for me!

DUK35

The weight gain that can be induced by insulin is a known concern and a common psychological barrier to treatment for some individuals. Several suggestions to mitigate this problem were proposed by members:

Post: Right, I really need to knock my eating back into shape again to get on top of this weight gain, what do you suggest?

Reply: The reason people put weight on when they start insulin is that they do not modify their diet...if you continue to eat lots...the weight will go on.

Other members offered alternative strategies:

Reply: One thing I have found helpful is intermittent fasting...it really works a treat.

Reply: I follow the MUFA diet—lots of dark chocolate, oils, olives, and nuts and seeds and avocadoes won’t hurt!

This led to gratitude, but some confusion:

Original poster: Thank you for all your interesting answers. I feel more positive about it, but it’s all quite confusing, I just don’t want to have this pot belly anymore!

DC10

Advice to increase physical activity levels is a core lifestyle recommendation in traditional diabetes care. However, barriers including affordability, acceptability, and conflicting advice regarding the need for and the type of physical activity in this group were illustrated in the threads:

Can’t afford a gym and would be too embarrassed to walk into one anyway!

DCUK10

Exercise is good and can help with insulin resistance, it isn’t necessarily needed for weight loss, it’s a must!

DCUK10

Exercise helps but the type of exercise if important [provides a list of weight bearing activity e.g., squats, lifting water bottles above head]

DCUK10

For me, running disappointingly sends my BG upwards, but a good brisk walk or working in the garden will almost always bring it down

DUK1

### Relationships With HCPs

Forum members also shared experiences of their relationships with HCPs. Many referred to the positive, if limited, access to the support of general practice or diabetes specialist nurses, who were evidently their preferred diabetes caregivers:

Post: Was doing fine on insulin for a couple of years and now its 69 (HbA1c) since my doctor changed the brand, should I just change back?

Reply: Best thing is to see your doctor, are you due to see him or her?

Reply: No, I but have been waiting to see my nurse for a while, I know if I explain it to her, she should be able to help me to make this work.

DCUK04

Furthermore, some members described negative experiences with HCPs. A paternalistic attitude appeared a common but unwelcome finding and was not appreciated, particularly when things were not going well:

I did as I was told, I was being a “good girl”, but my HbA1c was getting progressively worse

DCUK 31

It makes sense not to overload with too much information...but my view is we should be given all the information we need to manage our condition...rather than taking a paternalistic attitude and treating us like children.

DUK35

Lack of perceived HCP knowledge or expertise was also expressed:

Truth be told I never really understood diabetes before, but now I think I understand more [than their HCP] and it seems I haven’t been given great advice.

DUK10

In addition, a lack of educational provision and support was highlighted, reflecting the known inconsistency in care provision and “clinical inertia” in the United Kingdom:

Post: I have never been given any education or support, I diagnosed myself with diabetes after years of problems, no one even told me what kind of diabetes I had.

Reply: That is awful, please ask your GP for access to an education course... for type 2

DUK09

The apparent lack of confidence in HCPs translated into some members querying their diagnosis altogether, for example, through further tests, such as c-peptide, a blood test which indicates how much endogenous insulin (made by the body) a person is still producing, but which is not a routine diagnostic test for T2D in the United Kingdom:

So I had the test [c-peptide] privately, and it shows I do in fact have “robust levels of insulin” [ie, T2D].

DUK09

Finally, signposting to alternative and extended publicly available sources of information between members was used. For example, recommending links to YouTube videos, websites such as Bertie.org (carbohydrate counting), low carbohydrate diet pages, Diabetes.org, as well as an NHS helpline for insulin-treated adults with T2D. These strategies were perceived as more empowering and easily accessible than seeking help from existing health care provision.

### A Source of Psychological Peer Support

It was clear that many members felt unprepared to self-manage the practicalities of insulin treatment; however, unmet psychological factors were given equal weight.

Anxiety and emotional distress around insulin were common but countered by sharing reassuring and empowering messages from those who were already on established treatment:

Post: So scared and anxious to start insulin

Reply: I just dreaded the thought of insulin!...but don’t be scared...because it can really change your life for the better...once I was done with the initial phase, my BG [blood glucose] levels returned to somewhat normal and I literally felt amazing.

DUK15

Reply: Please try not to panic. I’ve been on insulin for a couple of years and tbh I am glad I am on it. Yes it’s a bit scary initially

DUK20

Other members were clearly fearful of injecting insulin and looking for reassurance and advice:

Post: I am really worried about getting the technique right [injection].

Reply: It won’t kill you or anything serious if you forget [the full technique]’ as long as the dose is right.

DUK20

Post: Where can I inject that won’t hurt, I’m scared to do this.

Reply: I find below the naval more comfortable and the outside of my thighs, just make sure to rotate [injection sites].

DUK20

In addition, sharing experiences of difficulties adjusting to insulin treatment elicited supportive responses:

Post: I am finding it difficult to get used to [taking insulin]...did it take people long?

Reply: I have been on it a few months and am still trying to figure it out...so yes it can take time to get right.

Reply: Thanks, good to know I’m not alone.

DUK10

## Discussion

### Principal Findings

For people with T2D who for whom insulin therapy is recommended or prescribed, web-based forums provide an opportunity to seek and receive advice, participate in discussions, and gain psychological support. Our analysis revealed that some individuals were struggling with basic unmet self-management needs such as injection technique. In addition, some were interested in extended self-management skills such as dose titration. However, giving titration advice was seen as out of bounds by some members, comparing it to prescribing and is a clear limitation of the forums. It was not evident whether members had been offered a one-to-one or structured group DSMES when starting insulin or ongoing support. However, the apparent need to seek alternative sources of information via the forum and elsewhere suggests that this is lacking or inconsistent for many. This finding is consistent with previous research findings from our team [19,27].

A new finding was the strong desire of the members to acquire extended advice about diet in relation to insulin treatment. Threads focused on achieving stable blood glucose levels and mitigating weight gain. A popular recommendation was a low or lower carbohydrate diet and in some cases carbohydrate counting. This may be unsurprising, as many of the responses were from people with T1D, who may be better educated and knowledgeable about the effects of carbohydrates on blood glucose levels. However, intermittent fasting and other weight management strategies were also topics of conversation. Although alternative diet strategies are currently popular in the media and are likely to be tried by many individuals, they are not routinely recommended or discussed as options in traditional health care provision. This is despite the National Institute of Health and Care Excellence and the current United Kingdom “evidence-based diet guidelines for the prevention and management of diabetes” [36] recommending that a personalized approach to diet choices, with ongoing support, is best practice. Our findings highlight that, although an alternative dietary approach is appealing to individuals in this group, appropriate dietary advice to support it is not available to most who receive routine care.

There was debate within the forum regarding the benefits of physical activity; some members lending it minimal importance, with others promoting the benefits and extolling the advantages of different forms; for example, weight bearing and aerobic. The current NHS recommendations for activity in adults are not condition-specific [37]. In addition, research on the benefits of different forms of exercise is currently emerging, particularly regarding weight loss and glycemia in T2D [37-39]. It appears that in this arguably motivated group, as with diet, there is a lack of appropriate education, information, or support available for individuals to make informed choices.

Some members recommended continuous glucose monitoring or Flash technology to others, despite it not being widely available to this group via the NHS [40]. However, there is a growing trend for self-funding within the T2D community, particularly in individuals who are insulin-treated. However, many HCPs responsible for diabetes care remain unfamiliar with emerging technology and are unable to advise on how to interpret it or use its full functionality. Issues around equity in health care provision regarding diabetes technology in the United Kingdom are not in the remit of this study, but are in need of further research in this group.

It was discouraging to discover continued reports of experiences of paternalistic attitudes from some HCPs, a phenomenon that has been documented for several decades [41]. Some members likened themselves to being made to feel like misbehaving children. This lack of trust and absence of a sense of equality in the HCP-patient relationship led some members to question their diabetes diagnosis altogether. Some sought private testing, which is not readily available via the NHS for people diagnosed with T2D; that is, c-peptide. It could be argued that this test is not necessary to make a clear diagnosis when an accurate history and full clinical picture is considered. However, the need for individuals to seek this reassurance outside of the NHS appears to reflect a lack of trust within it. This highlights the discrepancy that still appears to be present between best practice guidelines, such as the National Institute of Health and Care Excellence [42] and the American and European Diabetes Societies [43], all of whom advocate personalized care planning and support patient empowerment versus the realities of the clinical arena. This area warrants continuing close ethical scrutiny and the development of HCP educational strategies, incorporating awareness of the benefits of web-based forums for patients.

Underscoring the themes was the empowering nature of peer support. In addition to offering practical advice, usually based on members’ personal experiences, empathy and encouragement to counter negative cognitions were openly offered and exchanged. NHS England concurs that people living with similar conditions may feel connected to one another, and gaining support from people with direct relevant experience can enable them to manage their condition better [44]. Indeed, peer support has been identified by our team as an important aspect of group insulin DSMES [27]. Our current findings add a depth of understanding of the specific unmet needs that peers can help meet in this group, which has not been documented before.

### Strengths and Limitations

A growing, inclusive social media culture has enabled more individuals from diverse backgrounds to feel comfortable seeking and sharing advice and information on the web. By analyzing naturalistic interactions and conversations on the selected forums, additional insights into this field have been gained. There are potential limitations of this type of methodology, which we acknowledge. As highlighted previously, although 87% of adults use the internet and 72% of internet users have sought health information on the web, only 13% have posted on health-related forum [29]. Therefore, there is a risk that a few confident members, or “key players” posted on the forums, which may result in skewed data [45]. In addition, individuals who use forums may not be representative of the wider population. A lack of ability to count “views” (members reading a post or thread but not posting themselves) or the ability to collect detailed demographics are other limiting factors in this study.

### Implications

Internet-based research is an evolving field that serves to better understand the experiences and perspectives of individuals, which may not be included in traditional qualitative research methods, thus providing novel and rich data. The in-depth insights gathered from this study have provided a useful and valid contribution to the understanding of the experiences, perspectives, and unmet needs of individuals with T2D for whom insulin therapy is recommended, or prescribed.

### Conclusions

Our findings reveal that for people with T2D, health forums provide a rich source of self-management information while gaining psychological support, empathy, and encouragement from peers regarding insulin treatment. The forums also provide a safe space for individuals to express their frustration that these needs are not met through their usual health care provision, resulting in ambiguity over how to manage insulin effectively. This highlights a clear gap in the current health care provision in the United Kingdom for this group. The ultimate aim of our study was to enable individuals with T2D in this group to achieve their best outcomes. These new findings will directly contribute to the development of evidence-based insulin treatment, DSMES strategies, and raise awareness among HCPs and providers.

## Abbreviations

DSMES: diabetes self-management education and support
HbA1c: glycated hemoglobin
HCP: health care professional
NHS: National Health Service
T2D: type 2 diabetes
